# Effects of HIIT Interventions on Cardiorespiratory Fitness and Glycemic Parameters in Adults with Type 1 Diabetes: A Systematic Review and Meta-Analysis

**DOI:** 10.1007/s40279-024-02059-4

**Published:** 2024-06-21

**Authors:** Anja Lazić, Dušan Stanković, Nebojša Trajković, Cristina Cadenas-Sanchez

**Affiliations:** 1 Faculty of Sport and Physical Education, University of Niš, Niš, Serbia; 2 Department of Physical Education and Sports, Faculty of Sports Science, Sport and Health University Research Institute (iMUDS), University of Granada; CIBEROBN, ISCIII, Granada, Spain; 3 Stanford University, Department of Cardiology, Stanford; Veterans Affair Palo Alto Health Care System, Palo Alto, California USA

## Abstract

**Background:**

Individuals with type 1 diabetes mellitus (T1DM) face impaired cardiorespiratory fitness and glycemic control, increasing the risk of cardiovascular complications. High-intensity interval training (HIIT) has emerged as a promising exercise modality with potential benefits for both aspects in this population.

**Objectives:**

The primary aim was to investigate the effects of HIIT on cardiorespiratory fitness and glycemic parameters in patients with T1DM. The secondary aim was to examine the most effective HIIT protocol for cardiorespiratory fitness and glycemic parameters in patients with T1DM.

**Design:**

Systematic review and meta-analysis.

**Data Sources:**

Two major electronic databases (Web of Science and PubMed) were searched up to February 2024.

**Eligibility Criteria for Selecting Studies:**

Randomized and non-randomized trials involving adult patients with T1DM, free of complications and other diseases examining the effects of HIIT (HIIT pre vs. post; HIIT vs. control group or HIIT vs. moderate-intensity continuous training (MICT)) on cardiorespiratory fitness and glycemic parameters were included.

**Results:**

A total of ten studies met the inclusion criteria. The meta-analysis revealed a significant improvement in cardiorespiratory fitness following HIIT interventions (pre vs. post) in patients with T1DM (standardized mean difference (SMD) = 0.59, 95% confidence interval (CI) = 0.16 to 1, *p* = 0.01). Furthermore, HIIT (pre vs. post) was associated with significant improvements in 24-h mean glucose control (SMD = − 0.44, 95% CI = − 0.81 to − 0.06, *p* = 0.02), but the results (pre vs. post) failed to identify significant improvements in fasting glucose (SMD = − 0.26, 95% CI = − 0.78 to 0.24, *p* = 0.3) and glycated hemoglobin (HbA1C) values (SMD = − 0.28, 95% CI = − 0.61 to 0.05, *p* = 0.1). However, in comparison with a control group, HIIT showed significantly favorable effects on HbA1C (SMD = − 0.74, 95% CI = − 1.35 to − 0.14, *p* = 0.02). Finally, the meta-regression analysis did not find any moderating effect of any HIIT characteristics (i.e., intervention duration, session duration, work time, rest time, number of bouts, and intensity) on cardiorespiratory fitness and glycemic parameters.

**Conclusion:**

Our systematic review and meta-analysis show that T1DM patients who performed a HIIT intervention significantly improved cardiorespiratory fitness and reduced their 24-h mean glucose levels, but not their HbA1C and fasting glucose. These findings support the application of HIIT interventions in T1DM patients. However, the guidelines for the most effective protocol remain unclear; hence, future studies are needed.

**Supplementary Information:**

The online version contains supplementary material available at 10.1007/s40279-024-02059-4.

## Key Points

**Table:** 

HIIT has medium positive effects on cardiorespiratory fitness in adults with T1DM.
HIIT has a small positive effect on HbA1C and 24-h mean glucose in adults with T1DM.
Despite promising preliminary findings, the most effective protocol for improving cardiorespiratory fitness and glycemic control remains unclear due to limited research.

## Introduction

Type 1 diabetes (T1DM) is an autoimmune disease, with approximately 8.4 million diagnosed cases, affecting 5.4 million (64%) adults aged 20–59 years, and an alarming prediction of 13.5–17.4 million new cases by 2040 [1]. Given the early onset of T1DM in the pediatric population [2], the opportunities for disease prevention are limited. However, significant modifications and reductions in complications can be achieved [2]. Patients with T1DM face an increased risk of cardiovascular factors like obesity, hypertension, and hyperglycemia [3], attributed to impaired cardiorespiratory function, elevated glycated hemoglobin (HbA1C) levels, and impaired lipid status [4]. Consequently, cardiovascular disease is the primary cause of premature death in T1DM patients [5]. Therefore, effective prevention of complications and T1DM-related diseases is crucial for increasing the life expectancy of T1DM patients, emphasizing the significance of early intervention and comprehensive management strategies.

Regular exercise has received considerable research attention as an integral part of the treatment of T1DM [5–7]. Recent research [6, 8–11] confirms that regular exercise improves glucose metabolism, blood lipid status, and insulin sensitivity, while benefiting cardiorespiratory fitness [11], a strong prognostic risk factor for cardiovascular disease [12]. However, over 60% of adult T1DM patients do not engage in physical activity [13]. Previous studies [8, 13, 14] reported that patients with T1DM face numerous challenges and difficulties before, during, and after exercise sessions. The current guidelines [5, 15] strongly recommend regular exercise for patients with T1DM. However, patients face difficulties regarding prior carbohydrate intake according to exercise intensity and duration, glycemic monitoring, and a possible exercise-induced hypoglycemia [8, 14, 16]. Finally, there is disagreement on the safest and most effective exercise for T1DM patients [8, 17], with moderate-intensity continuous training (MICT) showing positive effects on cardiorespiratory fitness and HbA1C levels but causing hypoglycemia [8, 14, 17–19]. Contrarily, high-intensity interval exercise (HIIE) is associated with lower declines in glycemia [8, 20–22], albeit inducing transient hyperglycemic episodes (> 400 mg/dL) due to its anaerobic nature. It is important to note that cardiorespiratory fitness is a stronger factor in reducing the risk of cardiovascular disease than the general level of physical activity [23]. Therefore, improving cardiorespiratory fitness remains a key challenge, and high-intensity interval training (HIIT) is one of the most effective strategies [24]. Several authors [25–28] agree that HIIT can increase cardiorespiratory fitness in patients with T1DM. However, the debate on HIIT interventions’ impact on glycemic control persists. While some studies [29–31] suggested overall improvement in glycemic control, others [25, 26, 32] report no significant changes or even a slight and transient increase in blood glucose during exercise [33, 34]. The understanding of HIIT’s long-term effects on glucose variability is limited, emphasizing the need to prioritize feasible and effective exercise strategies while considering the complexities involved.

Therefore, the purpose of this work was to systematically review and meta-analyze all studies focused on the effects of HIIT on cardiorespiratory fitness and glycemic parameters in adults with T1DM. The secondary aim of this systematic review and meta-analysis was to determine the most effective HIIT protocol for cardiorespiratory fitness and glycemic control in adults with T1DM.

## Methods

### Protocol Registration

The updated guidelines of the Preferred Reporting Items for Systematic Reviews and Meta-Analyses (PRISMA) were used to conduct this systematic review and meta-analysis [35]. The review and meta-analysis protocol was registered with the International Prospective Register of Systematic Reviews (PROSPERO, Registration number: CRD42022371785).

### Data Source and Search Strategy

Two major electronic databases [i.e., Web of Science and Medical Literature Analysis and Retrieval System Online – MEDLINE (accessed by PubMed)] were searched from 2007 to 7 February 2024. We restricted the search strategy to the last 17 years to represent the most recent knowledge in the area of investigation as well as because of the increasing popularity of HIIT interventions in T1DM patients in the last decade. The complete search strategy used for each database is available in the Online Supplementary Material (OSM) Table S1.

### Eligibility Criteria

Two authors (A.L. and D.S.) independently assessed the eligibility criteria of the selected studies. The following inclusion criteria were based on the PICOT strategy (P –participants, I – Intervention; C—Comparison O—Outcome; T—time): (1) adults (18–65 years old) diagnosed with T1DM, free of complications or other diseases such as cardiovascular diseases, obesity, and other medical conditions; (2) randomized or non-randomized controlled intervention studies published in English in peer-reviewed journals with a Journal Citation Reports Index; (3) exercise intervention consisted of HIIT at least twice per week for a period longer than 3 weeks; (4) comparison groups included pre-intervention (baseline) values and/or control groups; and (5) outcome measures included at least one cardiorespiratory fitness parameter (i.e., maximal oxygen uptake (VO_2max_), peak rate of oxygen consumption (VO_2peak_)) and/or at least one glycemic control parameter (HbA1C, 24-h mean glucose and/or fasting glucose).

We excluded studies that combined HIIT interventions with other health interventions (e.g., dietary interventions), and ineligible publication types (e.g., reviews, editorials, commentaries, guidelines, cross-sectional or case reports).

### Study Selection

The selection process was conducted in three steps. First, two authors (A.L. and D.S.) independently identified and flagged potentially eligible studies by searching in Web of Science and PubMed. Second, after removing duplicates, potentially eligible studies were identified by reviewing the titles and abstracts. Finally, the full text of articles that appeared to be eligible was reviewed to determine whether or not they were eligible for further analysis. All data were extracted and imported into the “Rayyan” web-based tool for systematic literature screening. Independent literature review was done by two different authors (A.L. and D.S.). Any discrepancies identified were resolved by discussion of prior inclusion or exclusion criteria. When agreement could not be reached between the two authors, a third author (N.T.) was consulted. In addition, we manually reviewed all reference lists of previous systematic reviews or narrative reviews on T1DM patients and exercise to identify studies that we may have missed.

### Data Collection and Extraction Process

An EndNote library was created for data collection (Clarivate Analytics, New York, NY, USA). The extracted data from the included studies were: (1) study (first author's last name); (2) study design (randomized controlled trial (RCT) or not RCT); (3) sample size and percentage of female participants; (4) characteristics of the study sample (i.e., age (expressed as mean and standard deviation or range), weight status, medication); (5) intervention characteristics (i.e., intervention duration (weeks), frequency (times/week), session duration (min), work time (seconds), rest time (seconds), bouts (number), HIIT intensity, type of HIIT), and conditions (i.e., whether the diet was controlled, strategies before/during/after the intervention); (6) outcomes (cardiorespiratory fitness (VO_2max_ and VO_2peak_) and/or glycemic parameters (HbA1C, 24-h mean glucose and fasting glucose)); and (7) main findings. When data were graphically presented, we extracted data using WebPlotDigitizer online software. Data collection and extraction were independently double-checked by two authors (A.L. and D.S.).

### Risk of Bias Assessment

Risk of bias was assessed by two independent authors (A.L and D.S.). Disagreements were resolved in a consensus meeting, and any discrepancy was discussed and agreed upon with a third author (N.T.). The internal validity of the selected studies was assessed using the Physiotherapy Evidence Database (PEDro) [36]. The PEDro scale is an 11-item rating scale that includes information on eligibility, participant allocation, blinding, outcome measures, statistical comparisons, etc. The authors assigned a score of 0 (if criteria were not met; answer "no" response) and 1 (if criteria were met; answer "yes" response). Scores for each criterion were summed for all studies. Studies were classified as "poor" if scores were 0–3, "fair" if scores were 4–5, "good" if scores were 6–8, and ''excellent'' if scores were 9–10. A detailed description of the PEDro checklist can be found in OSM Table S2.

### Quantitative Evidence Synthesis: Meta-Analysis

Standardized mean differences (Hedges’ SMD) and 95% confidence intervals (CIs) were calculated. Moreover, SMDs of 0.2–0.49, 0.5–0.8, and > 0.8 were considered to represent small, medium, and large differences, respectively [37]. The *I*^2^ statistic was used to assess the degree of heterogeneity. Values from < 50% show low heterogeneity, 50–75% moderate heterogeneity, and values > 75% indicate a high level of heterogeneity. SMD was determined using random effects models, due to the inconsistency in experimental factors (e.g., length of intervention and type of HIIT). Funnel plots and Egger’s intercept were evaluated to assess the risk of potential publication bias [38]. Additionally, the “trim and fill” procedure [39] was performed to adjust for the suspected publication bias where the pooled SMD was recalculated to include hypothetical missing studies when necessary. Furthermore, a leave-one-out cross-validation analysis was performed to test the impact of exclusion on the combined SMD by omitting one study at a time.

The analyses were focused on examining the effect of HIIT intervention on cardiorespiratory fitness and glycemic outcomes in the following groups (when possible): (1) within-groups (pre vs. post), (2) HIIT versus control groups, and (3) HIIT versus MICT groups. Additionally, given the previous evidence might suggest a minimum of 12 weeks for significant reductions in HbA1C, we performed a sensitivity analysis excluding those studies with interventions of less than 8 weeks.

Furthermore, a random-effects meta-regression was performed to examine whether the effects of HIIT on cardiorespiratory fitness and glycemic parameters were moderated by different intervention-related characteristics (i.e., duration of intervention (weeks), frequency (times/week), session duration (minutes), work time (seconds), rest time (seconds), number of bouts, and intensity (% of HRmax)).

The meta-analysis was performed using the MedCalc Statistical Software version 19.6 (MedCalc Software Ltd, Ostend, Belgium). The meta-regression analysis was performed using the Comprehensive Meta-Analysis software (version 2.0; Biostat Inc., Englewood, NJ, USA). The statistical significance threshold was set at *p* < 0.05.

### Equity, Diversity and Inclusion Statement

The authors on this project were chosen on merit and came from a diverse range of backgrounds, occupations, and levels of seniority. We committed to promote diversity, equity, and inclusion in our clinical work, research, and training programs. We recognize the influence of systemic inequalities and biases on the research process, and we have worked to tackle these obstacles. As a result, we conducted data extraction, processing, and interpretation with minimal limitations to ensure that our findings reflect an accurate and inclusive representation of reality.

## Results

### Study Selection

**Fig. 1:**
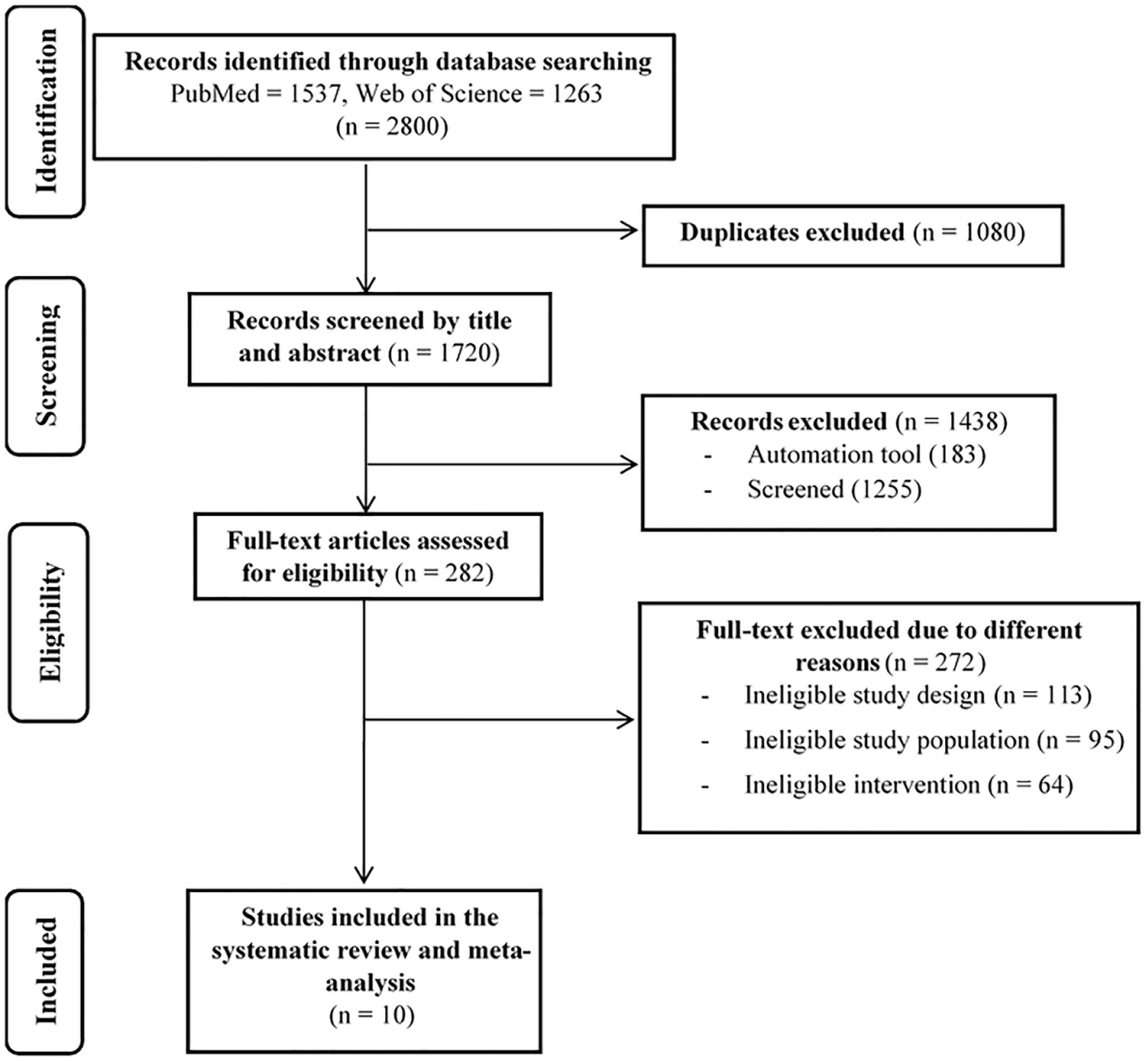
Studies included through the review process according to the Preferred Reporting Items for Systematic Reviews and Meta-Analyses (PRISMA) guidelines

The initial search identified 2800 articles from two databases, with 1720 records remaining after de-duplication. Title and abstract screening eliminated 1438 articles with assistance of an automation tool; 282 articles remained for full-text screening and eligibility. Of these 272 studies were excluded during the full-text screening for the following reasons: ineligible study design (*n* = 113), ineligible study population (*n* = 95), and ineligible intervention (*n* = 64). Therefore, a total of ten studies were included in the systematic review and meta-analysis: six RCTs and four non-RCTs. Details of study selection using the PRISMA flow diagram are shown in Fig. 1.

### Risk of Bias Assessment

The PEDro score indicated the good quality of eligible studies, with an overall mean of 6.2. All included studies met eligibility, intention-to-treat analysis, point estimates and variability criteria. However, low scores were obtained for blinding of participants (no studies met this criterion due to obvious reasons). Moreover, only one study [25] met the criteria for blinding the clinician, and two studies [25, 29] scored positively for blinding accessor. Also, six studies [25–27, 29, 31, 40] satisfied randomization and concealed allocation, whereas the criteria for baseline comparability and between-group analysis were not satisfied by two [28, 41] and only one study [42], respectively. Finally, seven studies [25–29, 31, 40] were recognized with good quality and only three studies [32, 41, 42] received fair quality ratings. Table S2 in the OSM shows the risk of bias results for each of the studies included.

### Data Extraction: Study Characteristics

#### Sample Characteristics

The total sample covered by this systematic review and meta-analysis consisted of 204 participants. The number of participants in individual studies ranged from six [41] to 30 [40]. In addition, one study [41] included an exclusively male population, whereas participants in other studies were composed of both men and women. Four studies [28, 32, 41, 42] had no control or comparison group, while six studies included a control group [25, 27, 31] or group that performed another intervention program (i.e., strength training (ST) [29], MICT [25, 26, 40], or a combination of ST and HIIT [29]). Table 1 summarizes the main characteristics of the included studies.

**Table 1:** Characteristics of the included studies (*n* = 10)

Study	Design	Sample size, groups (% F)	Characteristics of the study sample		
			Age (y)	Weight (kg)	Medication
Alarcón -Gomez et al. [27]	RCT	HIIT—11 (54.5%) CONT – 8 (50%)	HIIT—38 ± 5.5 CONT—35 ± 8.2	HIIT—70.5 ± 7.4 CONT—72.05 ± 5.0	Insulin injection
Boff et al. [25]	RCT	HIIT—9 (66.7%) MICT—9 (66.7%) CONT—9 (55.5%)	HIIT—26.1 ± 7.8 MICT—23.7 ± 5.8 CONT—20.8 ± 2.6	HIIT—64.1 ± 7.3 MICT—71.4 ± 11.6 CONT—65.6 ± 9.5	Insulin injection
Farinha et al. [29]	RCT	HIIT—9 (44.4%) ST—9 (44.4%) ST + HIIT -10 (50%)	HIIT—25.4 ± 5.6 ST—22.9 ± 3.6 ST + HIIT—24.9 ± 5.7	HIIT—67.1 ± 11.1 ST—64.8 ± 9.8 ST + HIIT—71.6 ± 13.6	Insulin injection or CSII
Lee et al. [31]	RCT	HIIT—12 (50%) CONT—15 (33.3%)	HIIT—40.5 ± 10.0 CONT—46.1 ± 10.5	HIIT—88.6 ± 10.0 CONT—92.2 ± 16.7	Insulin injection or CSII
Martín-San Agustín et al. [41]	Non RCT	HIIT—6 (0%)	36.7 ± 6.1	78.5 ± 6.1	Insulin injection
Minnebeck et al. [32]	Non RCT	NW—11 (45.5%) OW—11 (18.2%)	NW—42.18 ± 15.47 OW—40.73 ± 14.28	NW—74.42 ± 10.34 OW—94.95 ± 10.27	Insulin injection or CSII
Murillo et al. [40]	RCT	HIIT—17 (35.3%) MICT—13 (23.1%)	HIIT—35.4 ± 6.3 MICT -35.5 ± 12.2	HIIT—75.5 ± 20.1 MICT—72.9 ± 16.1	Insulin injection or CSII
Scott et al. [26]	Non RCT	HIIT—7 (28.6%) MICT—7 (28.6%)	HIIT—29 ± 3 CONT—29 ± 5	HIIT—90.0 ± 4.8 MICT—76.7 ± 5.4	Insulin injection
Scott et al. [42]	Non RCT	11 (63.6%)	30 ± 3		Insulin injection
Zinn et al. [28]	Non RCT	20 (30%)	41.3 ± 15.0	84.8 ± 14.7	Insulin injection or CSII

*CONT* control group, *CSII* subcutaneous insulin infusion, *F* female, *HIIT* high-intensity interval training group, *MICT* moderate-intensity continuous training group, *NA* not applicable, *NW* normal weight, *OW* overweight, *Non RCT* non-randomized controlled trial, *RCT* randomized controlled trial, *ST* strength training

#### Outcome Measure Characteristics

Cardiorespiratory fitness outcomes were reported using the VO_2peak_ parameter in five studies [25, 26, 29, 31, 42] and VO_2max_ in two studies [27, 40]. Moreover, outcome measures for glycemic control were obtained for HbA1C from six studies [25, 28, 29, 31, 32, 41], 24-h mean glucose from five studies [26, 31, 40–42], and fasting glucose from three studies [25, 27, 29]. The outcomes of the included studies are presented in Table 2.

**Table 2:** Characteristics of the high-intensity interval training (HIIT) interventions (*n* = 10)

Study	Diet	Strategies	Intervention duration (weeks)	Frequency (times/week)	Session duration (min)	Work time (s)	Rest time (s)	Number of bouts		Intensity	Activity	Main outcomes	
								Start	End			Cardiorespiratory fitness	Glycemic parameters
Alarcón -Gomez et al. [27]	Usual diet habits	15–30 g of CHO if glycemia values were < 5.6 mmol/L Small insulin dose was self-administered if glycemia was > 13.9 mmol/L	6	3	28–40	30	60	12	20	85% (HR_max_)	Cycle ergometer	VO_2max_ ↑*	Fasting glucose ↓*
Boff et al. [25]	NA	10 g glucose gels whenever blood glucose was ≤ 5.55 mmol/L; 20% decrease in insulin baseline dose	8	3	30–40	Ph1 – 1200 Ph2 – 60 Ph3—60	Ph1 –300 Ph2 – 300 (AR) Ph3 – 240 (AR)	1	6	50–80% (HR_max_)	Cycle ergometer	VO_2peak_ ↑*	HbA1C ↔ Fasting glucose ↔
Farinha et al. [29]	Usual diet habits	15–30 g of CH were ingested when glycaemia fell to ≤ 5.6 mmol/L	10	3	25	60	60 (AR)	10	10	90% (HR_max_)	Cycle ergometer	VO_2peak_ ↑*	HbA1C ↓* Fasting glucose ↓*
Lee et al. [31]	Usual diet habits	NA	12	3	33	240	180	4	4	85–95% (HR_max_)	Cycle ergometer/treadmill	VO_2peak_ ↓	HbA1C ↓* 24-h mean glucose ↓*
Martín-San Agustín et al. [41]	Usual diet habits	Orange juice (200 mL) was ingested if blood glucose was < 3.9 mmol/L	12	3	4–11	20	10	8	16	Max reps	Elastic band bodybuilding exercises		HbA1C ↓ 24-h mean glucose ↓
Minnebeck et al. [32]		No insulin adjustments	4	2	16–20	60	60 (PR)	4	6	All—out	Cycle ergometer		HbA1C ↓ (NW, OW)
Murillo et al. [40]	Usual diet habits	5–15 g of glucose tablets if glycemia before or during intervention were < 5.6 mmol/L	3	3	35	30	120 (AR)	10	10	100–120% (VO_2max_)	Cycle ergometer	VO_2max_ ↑	24-h mean glucose ↓*
Scott et al. [26]	Standardized diet (50% CHO; 30% fat; 20% protein)	10 g glucose gels if blood glucose was ≤ 5.55 mmol/L and a 20% decrease in insulin baseline dose was recommended to all individuals in the morning of every training day	6	3	15—23	60	60 (AR)	4	10	All—out	Cycle ergometer	VO_2peak_ ↑*	24-h mean glucose ↓
Scott et al. [42]	Self-recorded but usual dietary habits	NA	6	3	20	60	60 (PR)	6	6	> 80% (HR_max_)	18 random bodyweight exercises	VO_2peak_ ↑*	24-h mean glucose ↓
Zinn et al. [28]	Usual diet habits	No insulin adjustments but if glycaemia was between 5 and 15 mmol/L participants were suspended from training during 24 h	4	2	16–20	60	60 (PA)	4	6	All – out	Cycle ergometer		HbA1C ↓

*AR* active recovery, *CH* carbohydrates, *CHO* fast-acting carbohydrates, *HbA1C* glycated hemoglobin, *HR*_*max*_ maximal heart rate, *max reps* maximal number of repetitions, *NA* not applicable, *Ph* phase, *PR* passive recovery, *VO*_*2max*_ maximal oxygen uptake, *VO*_*2peak*_ peak rate of oxygen consumption, * statistically significant, ↑ improvement, ↓ reduction, ↔ unchanged

#### Intervention Characteristics

The total duration of intervention was as follows: 3 weeks [40], 4 weeks [28, 32], 6 weeks [26, 27, 42], 8 weeks [25], 10 weeks [29], and 12 weeks [31, 41]. The most common frequency of HIIT intervention was 3 days per week [25–27, 29, 31, 40–42]. Moreover, three studies [25, 29, 31] reported a duration of session lasting from 20 to 40 min. The reported duration of single sessions exhibited notable variation, ranging from 11 min [41] to 40 min [25, 27]. Intervention intensity ranged from 80% [25] of maximum heart rate to “all out’’ [26, 28, 32]. Eight studies [25–29, 31, 32, 42] reported intensity using percentage (%) of individual maximum heart rate, and there were two studies [40, 41] in which intensity was determined by maximal number of repetitions [41] and pecentage of VO_2peak_ [40]. The most common activity modality was cycle ergometer [25–29, 31, 32, 40], followed by treadmill [31], bodyweight training [42], and bodybuilding exercises with elastic bands [41]. The specific protocols of the HIIT interventions used in each study are presented in Table 2.

#### Diabetes Management

To prevent sudden glycemic changes throughout the HIIT, the following strategies were implemented: glucose and carbohydrate intake [25–27, 29, 40, 41], insulin adjustments [25–27], without insulin adjustments [28, 32], and strategy not specified [31, 42]. Table 2 summarizes the management strategies employed in each included study.

### Synthesis of Findings: Quantitative Evidence

#### Effect of HIIT on Cardiorespiratory Fitness in T1DM Patients

**Fig. 2:**
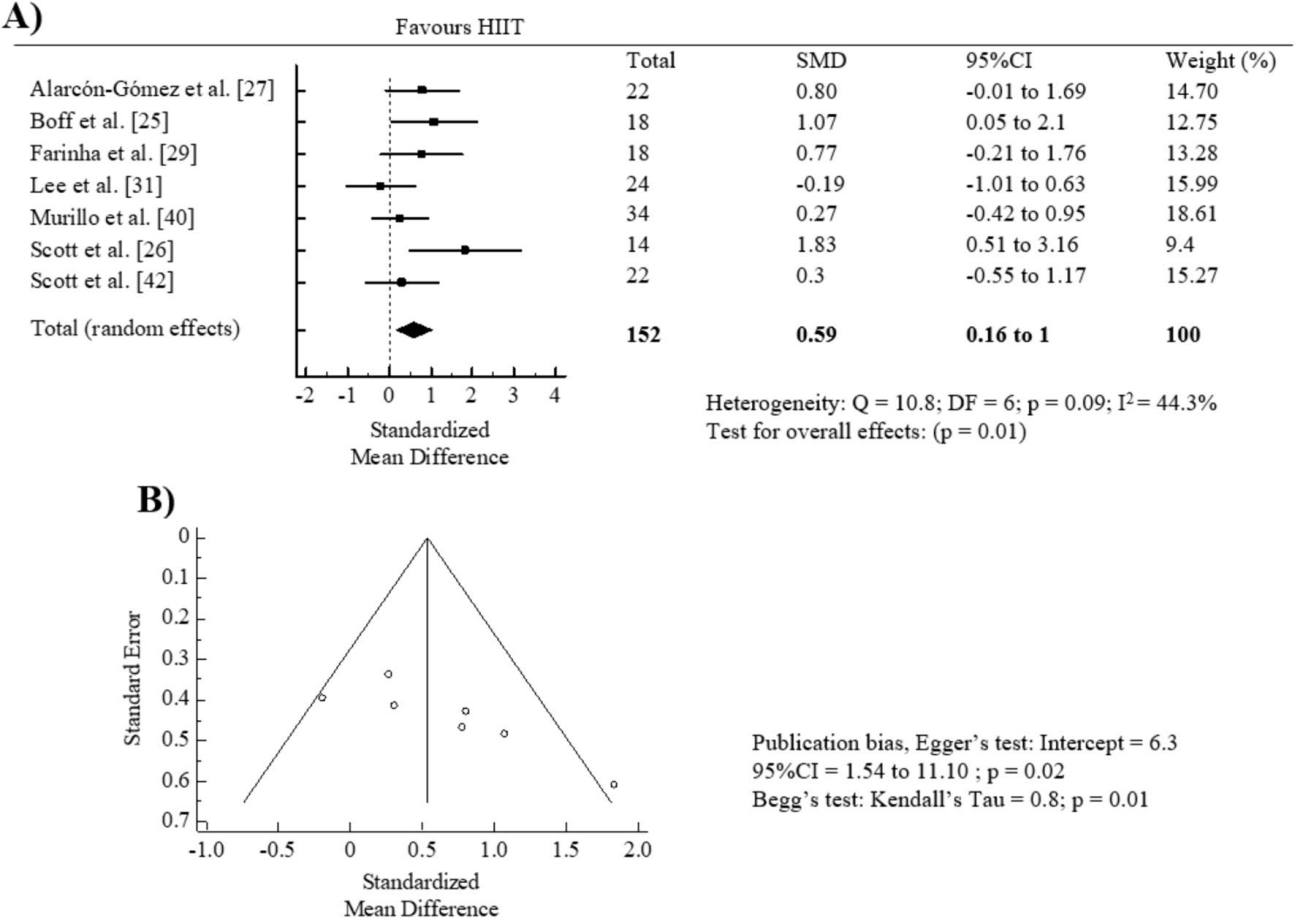
Forest plot of within-group effect (**A**) and publication bias (**B**) of high-intensity interval training (HIIT) on cardiorespiratory fitness

The meta-analysis of HIIT intervention (pre vs. post) on cardiorespiratory fitness showed a significant pooled SMD (SMD = 0.59, 95% CI = 0.16 to 1, *p* = 0.01; *I*^2^ = 44.3%) (Fig. 2A). There was a statistically significant publication bias according to Egger’s test (*p* = 0.02) or visual inspection of the funnel plot for cardiorespiratory outcomes (Fig. 2B). However, the “trim and fill” procedure showed that the estimates were not changed, and, thus, no correction for potential publication bias was needed. The leave-one-out analysis did not alter the results.

**Fig. 3:**
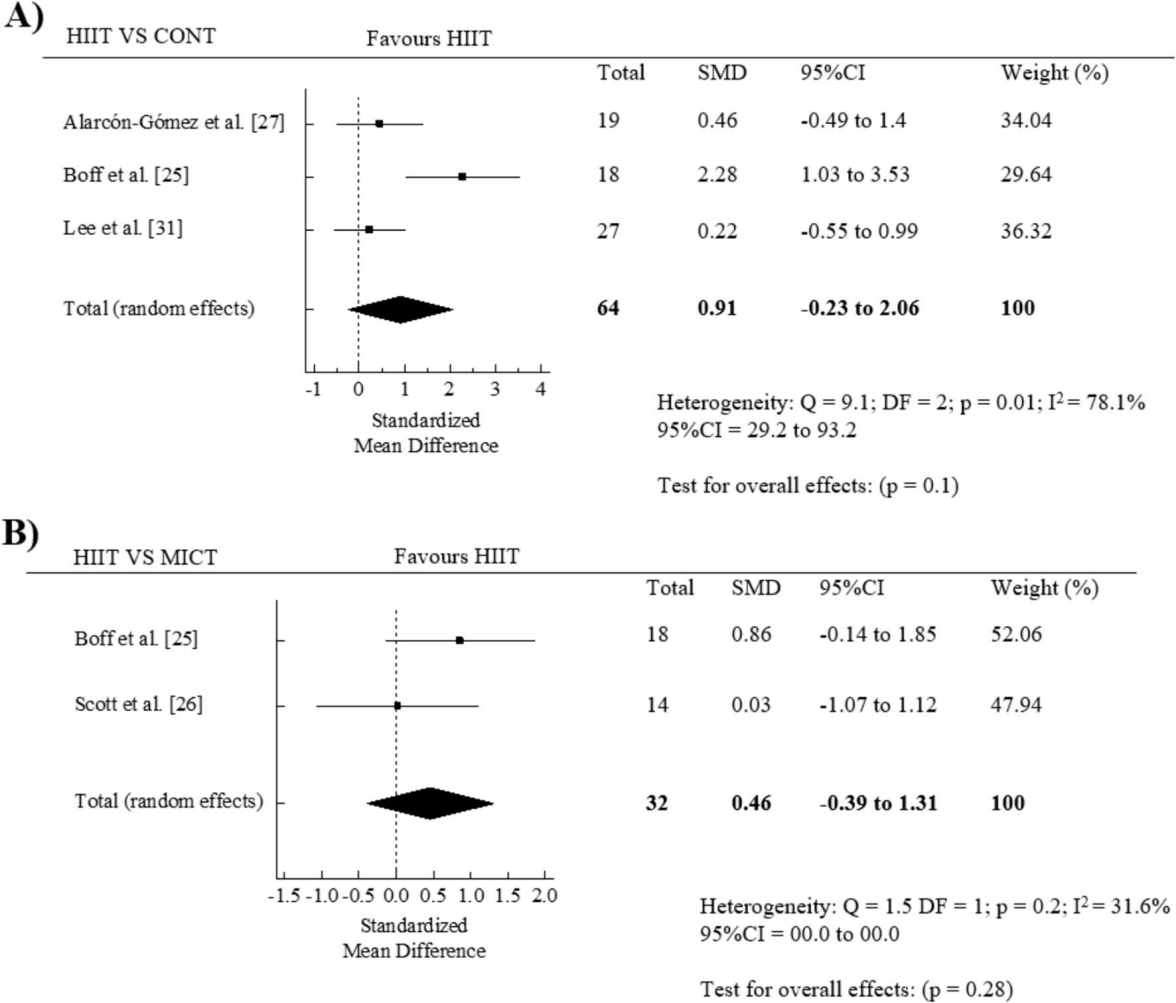
Forest plot for change in cardiorespiratory fitness between high-intensity interval training (HIIT) and control (CONT) groups (**A**), and between HIIT and moderate-intensity training (MICT) (**B**)

As exploratory analyses, we examined whether the effect of HIIT intervention differed in those studies that measured cardiorespiratory fitness only by VO_2peak_ (mL/kg/min) (Fig. S1, OSM) and VO_2max_ values (mL/kg/min) (Fig. S2, OSM). When comparing with pre vs. post intervention values, the SMD was 0.68 (95% CI = 0.03 to 1.32, *p* = 0.04; *I*^2^ = 58.9%) for VO_2peak_ and 0.47 (95% CI = − 0.06 to 1, *p* = 0.08; *I*^2^ = 0.00) for VO_2max_, respectively. Additionally, we further examined the effect of HIIT intervention on VO_2peak_ comparing the exercise versus control groups (*n* = 3 studies). The findings showed a non-significant pooled SMD for the efficacy of HIIT on cardiorespiratory fitness improvement (SMD = 0.91 in favor of HIIT, 95% CI = − 0.23 to 2.06, *p* = 0.1; *I*^2^ = 78.1%) (Fig. 3A). Finally, we also explored whether the differences in cardiorespiratory fitness (VO_2peak_) exist between HIIT versus MICT (*n* = 2 studies). The meta-analysis found a non-significant pooled SMD (SMD = 0.46 in favor of HIIT, 95% CI = − 0.39 to 1.31, *p* = 0.28; I^2^ = 31.6%) between the HIIT and MICT groups (Fig. 3B).

#### Effect of HIIT on Glycemic Parameters in T1DM Patients

##### HbA1C

**Fig. 4:**
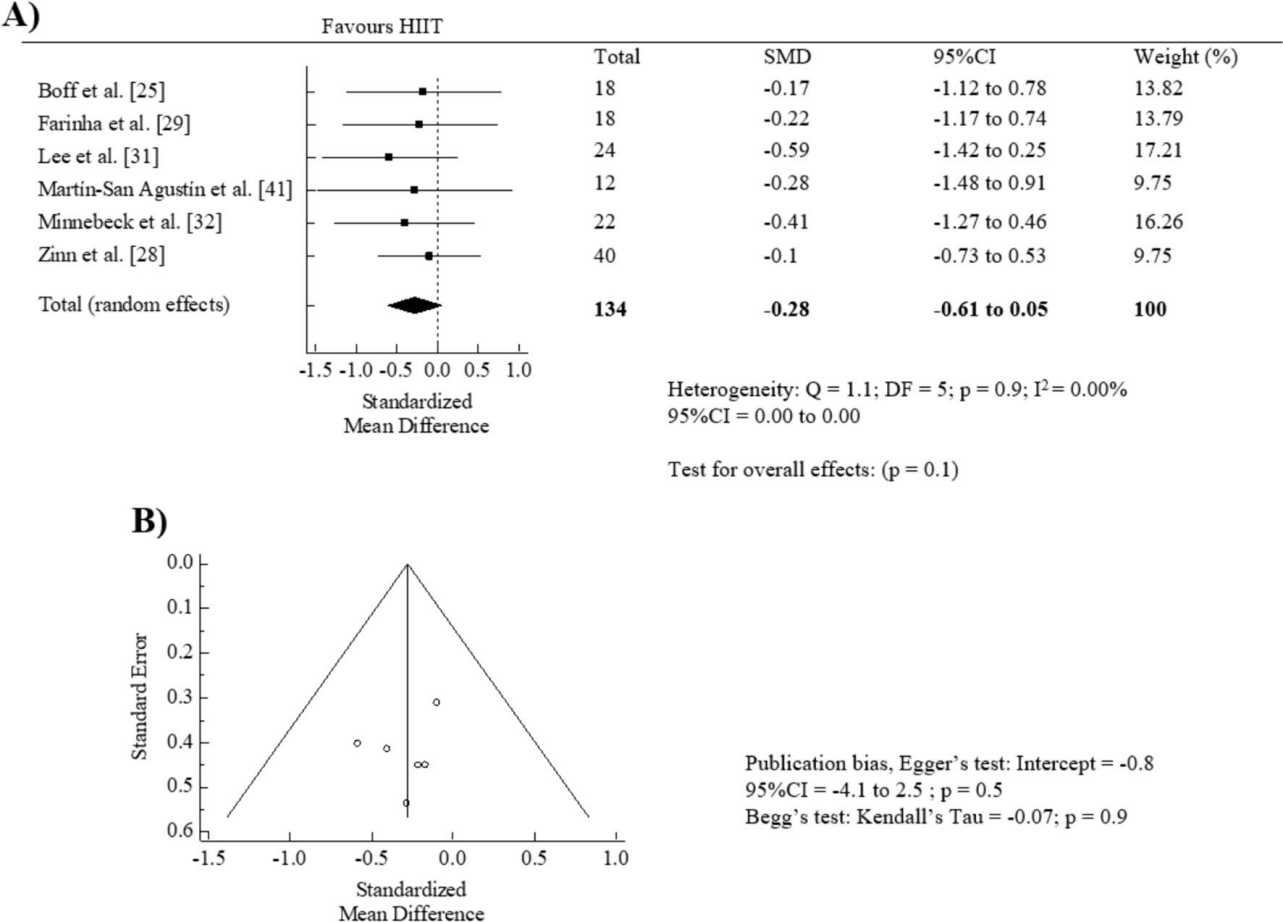
Forest plot of within-group effect (**A**) and publication bias (**B**) of high-intensity interval training (HIIT) on glycated hemoglobin (HbA1C)

The effect of HIIT intervention (pre vs. post) on HbA1C showed non-significant pooled SMD (SMD = − 0.28, 95% CI = − 0.61 to 0.05, *p* = 0.1; *I*^2^ = 0.00%) (Fig. 4A). No significant publication bias was detected with the Egger’s test (*p* = 0.5) and visual inspection of the funnel plot (Fig. 4B). The “trim and fill” procedure showed that the estimates were not changed. The leave-one-out analysis did not alter the results.

As exploratory analyses, we further examined whether the differences between HIIT and control groups exist for HbA1C (*n* = 2 studies). The pooled SMD was − 0.74 in favor of HIIT (95% CI = − 1.35 to − 0.14, *p* = 0.02; *I*^2^ = 0.00%) compared to the control group (Fig. S3, OSM). Moreover, only one study [25] examined the effect of HIIT versus MICT on HbA1C in T1DM patients, and consequently, we were unable to conduct a meta-analysis. Finally, a sensitivity analysis was performed excluding those studies (*n* = 2) with an intervention duration of less than 8 weeks. The pooled SMD was − 0.33 (95% CI = − 0.79 to 0.19, *p* = 0.15; *I*^2^ = 0.00%) (Fig. S4, OSM).

##### 24-h Mean Glucose Levels

**Fig. 5:**
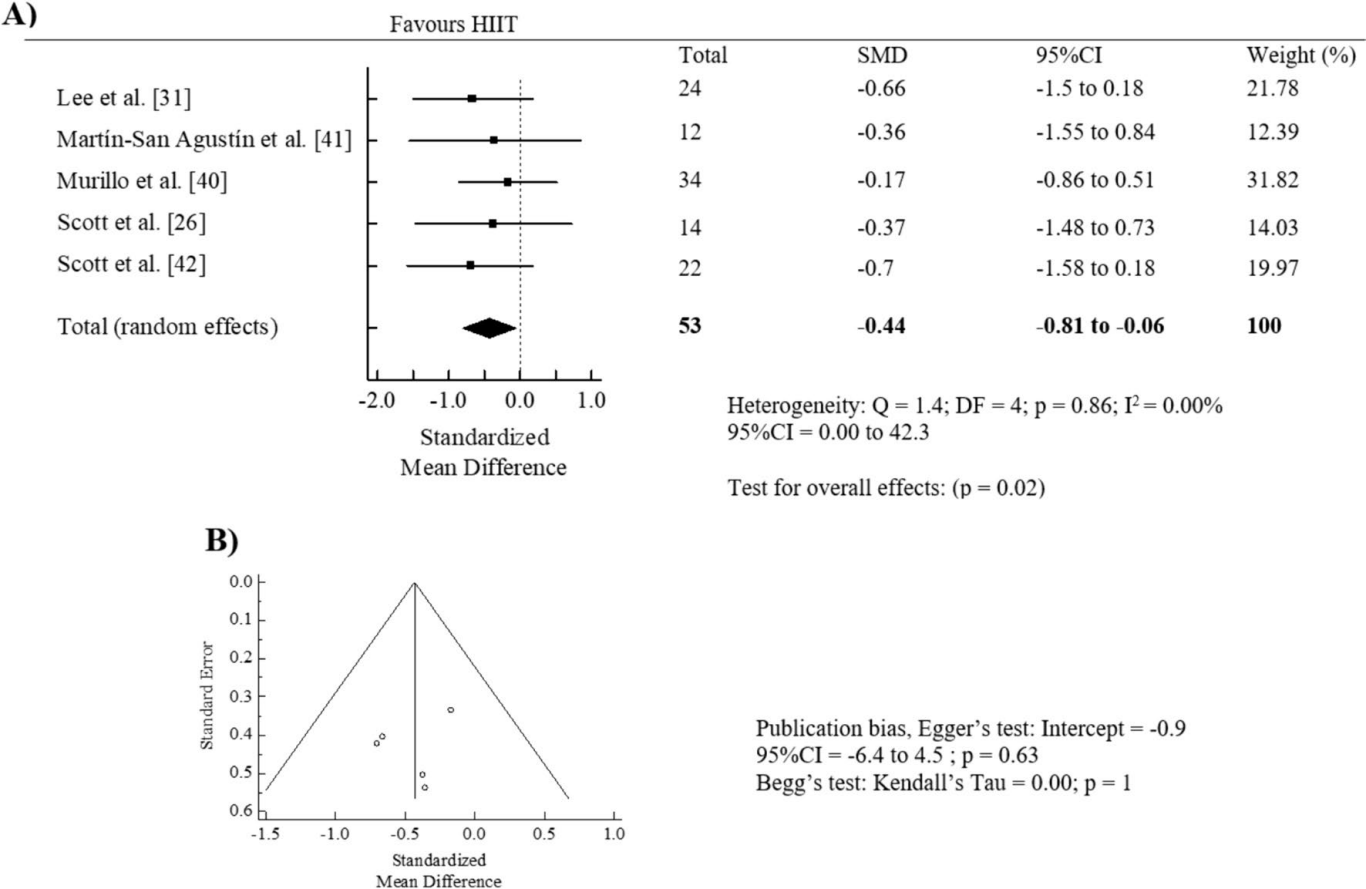
Forest plot of within-group effect (**A**) and publication bias (**B**) of high-intensity interval training (HIIT) on 24-h mean glucose levels

The effect of HIIT intervention (pre vs. post) revealed a significant pooled SMD reduction on 24-h mean glucose levels (SMD = − 0.44, 95% CI = − 0.81 to − 0.06, *p* = 0.02; *I*^2^ = 0.00%) (Fig. 5A). Egger's test (*p* = 0.6) and visual inspection of the funnel plot showed no significant publication bias for 24-h mean glucose levels (Fig. 5B). The “trim and fill” procedure showed that the estimates were not changed. The leave-one-out analysis did not alter the results. Due to the limited number of studies, we were unable to perform subgroup analyses on the effects of HIIT compared to the control groups.

##### Fasting Glucose

**Fig. 6:**
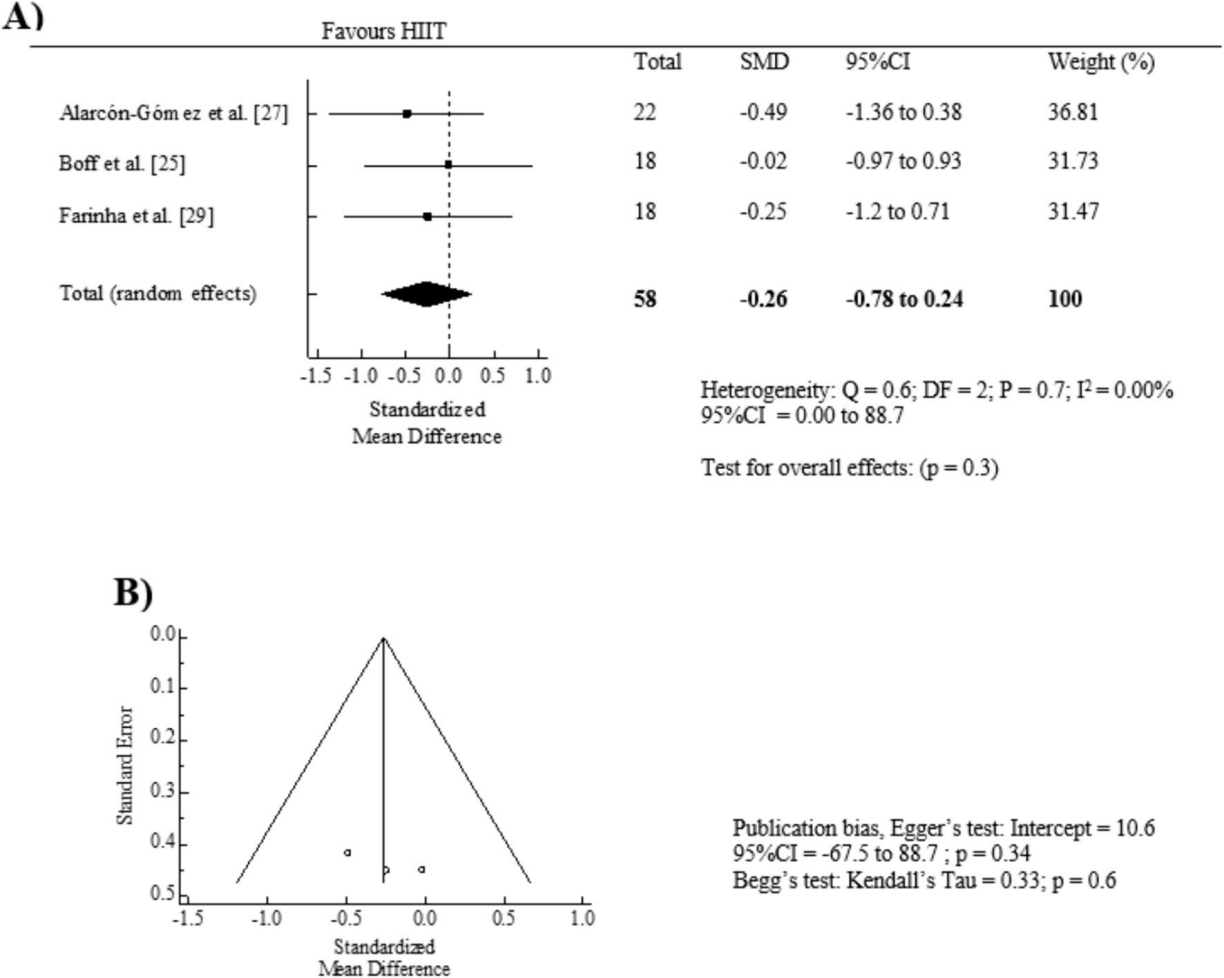
Forest plot of within-group effect (**A**) and publication bias (**B**) of high-intensity interval training (HIIT) on fasting glucose

The effect of HIIT intervention (pre vs. post) showed non-significant pooled SMD reduction on fasting glucose levels (SMD = − 0.26, 95% CI = − 0.78 to 0.24, *p* = 0.3; *I*^2^ = 0.00%) (Fig. 6A). Egger's test (*p* = 0.3) and visual inspection of the funnel plot showed no significant publication bias for fasting glucose levels (Fig. 6B). Additionally, the “trim and fill” procedure showed that estimates were not changed and the leave-one-out analysis did not alter the results. Furthermore, we examined whether the differences between HIIT and control groups exist for fasting glucose levels (*n* = 2 studies). The pooled SMD was − 0.27 in favor of HIIT (95% CI = − 0.89 to 0.35, *p* = 0.4; *I*^2^ = 0.00%) compared to the control groups (Fig. S5, OSM).

### Meta-Regression

Meta-regression showed that intervention duration (*β*: − 0.022; 95% CI = − 0.115 to 0.072; *p* = 0.646), session duration (*β*: 0.011; 95% CI = − 0.027 to 0.049; *p* = 0.566), work time (*β*: − 0.002; 95% CI = − 0.006 to 0.003, *p* = 0.445), rest time (*β*: 0.001; 95% CI = − 0.003 to 0.005; *p* = 0.631), number of bouts (*β*: 0.004; 95% CI = − 0.055 to 0.063; *p* = 0.895), and intensity (*β*: 0.005; 95% CI = − 0.033 to 0.043; *p* = 0.791) were not significantly associated with changes in cardiorespiratory fitness (Table S3, OSM). Similarly, changes in HbA1C were not influenced by intervention duration (*β*: 0.012; 95% CI = − 0.100 to 0.124; *p* = 0.837), session duration (*β*: 0.007; 95% CI = − 0.040 to 0.055; *p* = 0.769), work time (*β*: 0.004; 95% CI = − 0.005 to 0.014; *p* = 0.407), rest time (*β*: 0.001; 95% CI = − 0.004 to 0.006; *p* = 0.741), number of bouts (*β*: − 0.017; 95% CI = − 0.124 to 0.090; *p* = 0.757), and intensity (*β*: − 0.001 95% CI = − 0.050 to 0.047; *p* = 0.953) (Table S4, OSM). Finally, meta-regression analyses revealed no significant effects of intervention duration (*β*: − 0.035; 95% CI = − 0.134 to 0.064; *p* = 0.488), session duration (*β*: − 0.013; 95% CI = − 0.040 to 0.067; *p* = 0.624), work time (*β*: − 0.002; 95% CI = − 0.006 to 0.003; *p* = 0.457), rest time (*β*: − 0.001; 95% CI = − 0.007 to 0.006; *p* = 0.900), number of bouts (*β*: 0.041; 95% CI = − 0.059 to 0.141; *p* = 0.421), and intensity (*β*: 0.013; 95% CI = − 0.040 to 0.067; *p* = 0.624) with changes in 24-h mean glucose levels (Table S5, OSM). Similarly, no significant effects were observed for intervention duration (*β*: 0.064; 95% CI = − 0.236 to 0.365; *p* = 0.675), session duration (*β*: − 0.001; 95% CI = − 0.073 to 0.070; *p* = 0.969), work time (*β*: 0.012; 95% CI = − 0.022 to 0.046; *p* = 0.497), rest time (*β*: 0.002; 95% CI = − 0.004 to 0.008; *p* = 0.507), number of bouts (*β*: − 0.032; 95% CI = − 0.114 to 0.051; *p* = 0.453), and intensity (*β*: − 0.023; 95% CI = − 0.148 to 0.101; *p* = 0.715) regarding changes in fasting glucose levels (Table S6, OSM). Given the limited variability in intervention frequencies across the ten studies, with only two studies employing a different frequency, we were unable to conduct the analyses based on frequency.

## Discussion

The main findings of the meta-analysis are the following: (1) T1DM patients who performed a HIIT intervention significantly improved cardiorespiratory fitness; (2) T1DM patients significantly reduced 24-h mean glucose levels but not HbA1C and fasting glucose after a HIIT intervention; and (3) none of the HIIT characteristics (i.e., intervention duration, session duration, work time, rest time, number of bouts, and intensity) influenced improvements in cardiorespiratory fitness and glycemic control. To the best of our knowledge, this is the first systematic review and meta-analysis assessing the effects of HIIT intervention on cardiorespiratory fitness and glycemic outcomes in T1DM patients. Further, it is important to note that further RCTs are needed to strengthen the evidence base and provide more comprehensive insights into the impact of HIIT on cardiorespiratory fitness and glycemic outcomes in T1DM patients.

### Effect of HIIT on Cardiorespiratory Fitness in T1DM Patients

The existing scientific literature has highlighted the positive impact of exercise on cardiorespiratory fitness in individuals with T1DM [11, 17, 43]. However, there is a lack of meta-analyses specifically examining the effects of HIIT on cardiorespiratory fitness parameters in adults with T1DM.

Our meta-analysis findings indicate that HIIT interventions have a positive effect on cardiorespiratory fitness in adults with T1DM. Specifically, most of the studies included (*n* = 5) [25–27, 29, 42] showed a remarkable improvement in cardiorespiratory fitness by 11% with HIIT interventions, primarily using cycling as the exercise modality. In contrast, only one study [31] reported no improvements in cardiorespiratory fitness despite three sessions of weekly HIIT. This lack of improvement could be attributed to low adherence and poor compliance with use of the face mask during measurements. Furthermore, the subgroup analyses showed a non-significant trend towards improved cardiorespiratory fitness in the HIIT groups compared to both control and MICT groups. The observed discrepancy in the effect of HIIT on cardiorespiratory fitness between pre-post and control group could be attributed to several factors, including individual variability in response to HIIT, baseline cardiorespiratory fitness levels, and characteristics of HIIT. First, in pre-post studies, these individual variations might be averaged out, showing a general trend of improvement. Second, baseline cardiorespiratory fitness levels in individual studies were similar between the control and HIIT groups. Consequently, the intensity and volume may not have provided enough stimulus to elicit distinct improvements in cardiorespiratory fitness compared to the control group. Third, the control groups were advised to have their usual activities, which might increase their physical activity and improve general fitness, contributing to increased cardiorespiratory fitness even without a substantial VO_2peak_ change. In addition, it is important to highlight that the analysis was based on a relatively small number of interventions and participants, which may have affected the ability to detect subgroup differences. Finally, a high level of heterogeneity was observed in the HIIT versus control subgroup analysis, further impacting the validity of the treatment effect estimate.

Specifically, improving VO_2max_ through regular exercise has been established as achievable in adults with T1DM [44]. HIIT has been shown to be an effective way to increase VO_2max_ in patients with type 2 diabetes (T2DM) [45]. Our meta-analysis included only two studies that investigated the impact of HIIT on VO_2max_ in T1DM patients, with one study indicating negligible improvement after a 3-week intervention [40] and the other reporting a substantial 8.9% improvement following a 6-week HIIT protocol [27]. These findings suggest that the duration of the intervention might be an important factor in observing cardiorespiratory fitness improvements in T1DM patients through HIIT interventions. This hypothesis is further supported by the meta-regression analysis, where duration trended towards a moderating effect on cardiorespiratory fitness, despite a non-significant influence. However, due to the limited number of studies and participants, caution should be exercised when interpreting these conclusions.

Overall, this systematic review and meta-analysis suggests that HIIT interventions can positively impact cardiorespiratory fitness in adults with T1DM. However, the limitations of the included studies and the heterogeneity observed highlight the need for additional research to further elucidate the effects of HIIT on cardiorespiratory fitness in this population.

### Effect of HIIT on Glycemic Parameters in T1DM Patients

#### HbA1C

The findings of our meta-analysis indicate a non-significant effect of HIIT on HbA1C levels when compared to baseline measurements in adults with T1DM. However, a significant decrease in HbA1C levels was observed when comparing the HIIT group to the control group. It is important to interpret these results cautiously due to the limited number of RCTs available for analysis.

Existing studies examining exercise interventions and HbA1C levels in T1DM patients have provided contradictory findings. While our results align with some previous studies that did not show changes in HbA1C levels with exercise interventions [11, 17, 46], other meta-analyses have reported small reductions in HbA1c levels [2, 8, 43]. It is worth noting that none of these influential studies specifically focused on the effects of HIIT in T1DM patients, as HIIT has only recently gained popularity in this population.

Interestingly, the duration of HIIT interventions appears to be a key factor in influencing HbA1C levels. Our analysis indicates that short-term HIIT interventions may not be sufficient to significantly improve HbA1C levels, considering the lifespan of erythrocytes and the time required for meaningful changes to occur [11]. Previous research has suggested that a minimum duration of 12 weeks may be necessary for visible reductions in HbA1C levels [8]. However, we additionally evaluated those studies with a minimum duration of 8 weeks, and the results remained similar compared to those of 4 weeks. Further studies are needed to determine the duration of the intervention needed for HIIT to show a noticeable effect on HbA1C levels. Furthermore, a related idea that could elucidate the challenges associated with improving HbA1C across various training regimes may be attributed to the strategies developed before, during, or after the intervention (e.g., insulin adjustment, increased carbohydrate intake, or dietary control) to mitigate hypoglycemia [46]. However, despite meticulously adjusting insulin and carbohydrate intake, HbA1C improvements in individuals with T1DM after HIIT remain elusive due to multiple factors. Increased concentrations of counter-regulatory hormones, individual insulin sensitivity variations as well as baseline HbA1C (< 8%) may blunt improvements [8]. Additionally, studies included in our meta-analysis reported that diet was not controlled and individuals were advised to maintain their usual diet habits throughout the whole experimental period, which also may affect the lack of improvement in HbA1C. Certainly, the feasibility of maintaining a controlled diet requires careful consideration due to the inherent challenges of real-world settings.

Despite the non-significant changes observed in HbA1C levels, it is noteworthy that most individual studies reported lower HbA1C levels than baseline, indicating the potential effectiveness of HIIT in improving glycemic control. The mechanisms underlying these effects may be attributed to the noticeable improvements in cardiorespiratory fitness achieved through HIIT, which leads to increased muscle oxidative capacity and enhanced mitochondrial density [47]. Both of these factors have been identified as strong predictors of better glycemic control [48, 49]. Lastly, it should be mentioned how HIIT affects HbA1C compared to other training regimes. While ST primarily achieves HbA1C reductions through increased muscle mass and subsequent higher glucose uptake by skeletal muscles [8], HIIT shares some similarities with MICT, but differences occur due to its higher intensity. First, higher intensity elicits increased excess post-exercise oxygen consumption (EPOC) [50], leading to greater calorie expenditure contributing to better insulin sensitivity and glucose uptake [51]. Second, a potential contributor to improved HbA1C with HIIT could be changes in body composition (i.e., reduced fat mass and waist circumference) [51]. These changes are influenced by both the training and the changes in appetite regulation. HIIT has been shown to chronically decrease ghrelin and increase satiety hormones (GLP-1 and peptide Y) [52]. While we propose this mechanism, our study did not directly assess body composition changes, making it difficult to confirm these previous findings. Future research investigating how HIIT alters these factors in T1DM patients is crucial to fully understand its impact on HbA1C.

Overall, while our meta-analysis did not find a significant effect of HIIT on HbA1C levels in adults with T1DM, it is important to consider the limitations of the available evidence and the need for further research. Future studies should explore the optimal duration and intensity of HIIT interventions.

#### 24-h Mean Glucose and Fasting Glucose

Our meta-analysis depicted a small reduction in 24-h mean glucose levels after a HIIT intervention in adults with T1DM, but did not reveal a significant reduction in fasting glucose. This finding suggests that hyperglycemia experienced during intense exercise is a transient response to neuroendocrine and hormonal changes, and blood glucose levels gradually stabilize and even decrease over time. Previous research supports the notion that hyperglycemia primarily occurs during the early recovery period (1–2 h) in T1DM patients [53, 54]. Since lowering blood glucose levels is a key goal in T1DM management, it is reassuring that intense exercise does not lead to negative consequences of hyperglycemia or late hypoglycemia, as reported elsewhere [55].

Our meta-analysis provides encouraging evidence for the implementation of HIIT as chronic training in reducing blood glucose levels. Notably, our meta-analysis employed two blood glucose parameters: 24-h mean glucose and fasting glucose. While both parameters are crucial for glycemic control, they reflect distinct aspects of glucose regulation. This distinction necessitates a more nuanced discussion of the findings. Specifically, 24-h mean glucose levels provide a more comprehensive assessment of glycemic fluctuations throughout the day. This is particularly important because higher 24-h glucose levels have been linked to an increased risk of cardiovascular diseases [56]. Our findings align with those of a previous meta-analysis [57] showing that exercise led to a small but significant reduction in 24-h mean glucose. These changes may be attributed to the influence of confounding factors, such as dietary control, sex, and intensity [57]. Regrettably, a detailed exploration of these potential explanations falls outside the purview of our study, and future investigations should prioritize disentangling the influence of confounding factors.

In contrast, our findings are consistent with previous meta-analyses [58, 59] that reported no reductions in fasting glucose following HIIT in patients with T2DM. More precisely, fasting glucose reflects glucose levels after an overnight fast and it is a marker of hepatic insulin sensitivity [60]. Since fasting hyperglycemia is associated with elevated hepatic insulin resistance [61], and the liver contributes approximately 25% of postprandial glucose uptake, skeletal muscle remains the primary tissue responsible for the remaining glucose disposal [62]. Furthermore, previous studies [60, 63] have demonstrated that neither HIIT nor exercise at lower intensities enhance peripheral insulin sensitivity without impacting hepatic insulin sensitivity or endogenous glucose production. Consequently, these observations suggest that HIIT may exert a more significant influence on 24-h mean glucose levels compared to fasting glucose. Supporting this notion, Liubaoerjijin et al. [59] and Cassidy et al. [60] demonstrated that dietary restriction and body fat loss play a more prominent role in improving fasting glucose. While body composition parameters were not included in our analysis, individual studies have demonstrated a reduction in fat mass and increased lean muscle mass. This finding warrants further investigation to elucidate the potential relationship between HIIT interventions and body composition in patients with T1DM**.** However, it is important to note that there is limited research specifically investigating 24-h mean glucose levels and fasting glucose compared to control groups and other exercise programs with diversity in the duration of the interventions and methods used. Therefore, caution should be exercised in interpreting our findings.

Finally, several mechanisms might explain why HIIT has a positive and potentially greater effect on reducing blood glucose levels compared to other exercise types such as MICT or RT. Firstly, HIIT can enhance insulin sensitivity through increased activity of AMP-activated protein kinase (AMPK) and rapid translocation of glucose transporter type 4 (GLUT4) across the cell membrane (up to 260%) [64]. Secondly, this process primarily occurs in the muscle fibers that are predominantly engaged during HIIT, leading to increased capillarization and glucose uptake in those muscles. Finally, hepatic adaptations may also contribute to lower blood glucose levels, although these findings from Heled et al. [65] were conducted in vitro and cannot be generalized to the population at large.

Therefore, our meta-analysis demonstrates a small, but notable reduction in 24-h mean glucose levels following HIIT interventions in adults with T1DM. However, further research is needed to explore the effects of longer interventions and consider different parameters and methods for measuring blood glucose levels. The mechanisms underlying the beneficial effects of HIIT on blood glucose reduction warrant further investigation.

### Strengths and Limitations

To the best of our knowledge, this is the first systematic and meta-analysis providing the efficacy of HIIT on cardiorespiratory fitness and glycemic parameters such as HbA1C, 24-h mean glucose levels, and fasting glucose in adults with T1DM. Furthermore, the use of two major databases for the search and the extensive analysis (study selection, data collection, risk of bias assessment) by two independent researchers are other strengths to consider. The major limitation of this study is that the findings should be interpreted with caution due to the small number of trials and participants included in some subgroup analyses. Further, some methodological issues such as differences in the measurement of blood glucose across the studies need to be acknowledged as a limitation. Additionally, the wider range of heterogeneity levels observed in the meta-analysis means the findings need to be considered with caution. Lastly, despite several conducted meta-regressions, not a single moderator was found to be significant. The examination of a large number of intervention characteristics (i.e., intervention duration, session duration, work time, rest time, number of bouts intensity, modality) combined with the relatively small number of studies prevented us from conclusively identifying the most effective HIIT protocol for improving cardiorespiratory fitness and glycemic control.

## Conclusion

Our systematic review and meta-analysis show that T1DM patients who performed a HIIT intervention significantly improved cardiorespiratory fitness and reduced their 24-h mean glucose levels but not their HbA1C and fasting glucose. Moreover, HIIT had no beneficial effect on cardiorespiratory fitness compared to MICT. Lastly, contrary to our expectations, there was no impact of HIIT characteristics (i.e., intervention duration, session duration, work time, rest time, number of bouts, and intensity) on cardiorespiratory fitness and glycemic parameters. Nevertheless, the limited number of studies and sample size precludes definitive conclusions. This review offers promising evidence suggesting the potential benefits of HIIT for T1DM patients. However, guidelines for the most effective protocol, duration of activity, and type of activity still remain unclear. Future studies should continue to explore the effects of HIIT in T1DM patients through more RCTs, larger sample sizes, and longer durations. Additionally, consideration of individual patient characteristics, constant monitoring of blood glucose levels, adequate nutrition, and optimal insulin dosing are crucial when implementing HIIT protocols. Moreover, exploring sex differences after HIIT interventions in patients with T1DM will contribute to a more comprehensive understanding of sex-specific mechanisms, with broader implications for T1DM management.

## Supplementary Information

Below is the link to the electronic supplementary material.

Supplementary file1 (DOCX 210 KB)

## Data Availability

The original contributions presented in the study are included in the article and/or supplementary material. The raw dataset can be shared following reasonable request to the authors.
