# Supplementary material for: Effects of HIIT Interventions on Cardiorespiratory Fitness and Glycemic Parameters in Adults with Type 1 Diabetes: A Systematic Review and Meta-Analysis
Source: Sports Med. 2024 Jun 21;54(10):2645–61. doi: 10.1007/s40279-024-02059-4 (PMC11466984; doi:10.1007/s40279-024-02059-4)
Supplement: Supplementary file 1 — Supplementary file1 (DOCX 210 KB) [file 40279_2024_2059_MOESM1_ESM.docx]

**Supplementary material**

**Table S1.** Search strategy used in Web of Science and PubMed.

("Diabetes 1" OR "diabetes type 1" OR "type 1 diabetes" OR T1D) AND ("glycemic parameter" OR glycemic OR glycaemia OR glycemia OR "glycemic variability" OR "glycemic control" OR "blood-sugar" OR "cardiorespiratory fitness" OR "cardiopulmonary fitness" OR "cardiovascular fitness" OR fitness OR exercise OR CRF OR VO2max OR VO2peak OR "maximal oxygen consumption") AND (HIIT OR "high-intensity interval training" OR high-intensity OR interval training AND effect* OR intervention*) NOT "diabetes type 2" NOT "type 2 diabetes" NOT "diabetes 2" NOT review NOT animal NOT cancer

**Table S2.** PEDro scores of included studies.

|  | Criterion | | | | | | | | | | | |  |
| --- | --- | --- | --- | --- | --- | --- | --- | --- | --- | --- | --- | --- | --- |
| Study | 1 | 2 | 3 | 4 | 5 | 6 | 7 | 8 | 9 | 10 | 11 | ∑ | Qualitative evaluation |
| Alarcón-Gómez et al. [27] | Y | Y | Y | Y | N | N | N | N | Y | Y | Y | 6 | Good |
| Boff et al. [25] | Y | Y | Y | Y | N | Y | Y | N | Y | Y | Y | 8 | Good |
| Farinha et al. [29] | Y | Y | Y | Y | N | N | Y | Y | Y | Y | Y | 8 | Good |
| Lee et al. [31] | Y | Y | Y | Y | N | N | N | Y | Y | Y | Y | 7 | Good |
| Marin-San Agustin et al. [41] | Y | N | N | N | N | N | N | Y | Y | Y | Y | 4 | Fair |
| Minnebeck et al. [32] | Y | N | N | Y | N | N | N | Y | Y | Y | Y | 5 | Fair |
| Murillo et al. [40] | Y | Y | Y | Y | N | N | N | Y | Y | Y | Y | 7 | Good |
| Scott et al. [26] | Y | Y | Y | Y | N | N | N | Y | Y | Y | Y | 7 | Good |
| Scott et al. [42] | Y | N | N | Y | N | N | N | Y | Y | N | Y | 4 | Fair |
| Zinn et al. [28] | Y | N | N | N | N | N | N | Y | Y | Y | Y | 6 | Good |

Criterion 1—eligibility criteria; 2—random allocation; 3—concealed allocation; 4—baseline comparability; 5—blind subject; 6—blind clinician; 7—blind assessor; 8—adequate follow-up; 9—intention-to-treat analysis; 10—between-group analysis; 11 —point estimates and variability; Y—criterion is satisfied; N—criterion is not satisfied; ∑—total awarded points.

**Table S3.** Meta-regression of changes in cardiorespiratory fitness.

| **Covariate** | **Coefficient**  **(β)** | **95% CI** | **Standard error** | **p value** |
| --- | --- | --- | --- | --- |
| Intervention duration (weeks) | -0.022 | -0.115 to 0.072 | 0.048 | 0.646 |
| Session duration (min) | 0.011 | -0.027 to 0.049 | 0.019 | 0.566 |
| Work time (s) | -0.002 | -0.006 to 0.003 | 0.002 | 0.445 |
| Rest time (s) | 0.001 | -0.003 to 0.005 | 0.002 | 0.631 |
| Number of bouts | 0.004 | -0.055 to 0.063 | 0.030 | 0.895 |
| Intensity | 0.005 | -0.033 to 0.043 | 0.019 | 0.791 |

β – beta coefficient; 95% CI – confidence interval.

**Table S4.** Meta-regression of changes in glycated hemoglobin (HbA1C).

| Covariate | Coefficient  (β) | 95% CI | Standard error | p value |
| --- | --- | --- | --- | --- |
| Intervention duration (weeks) | 0.012 | -0.100 to 0.124 | 0.057 | 0.837 |
| Session duration (min) | 0.007 | -0.040 to 0.055 | 0.024 | 0.769 |
| Work time (s) | 0.004 | -0.005 to 0.014 | 0.005 | 0.407 |
| Rest time (s) | 0.001 | -0.004 to 0.006 | 0.002 | 0.741 |
| Number of bouts | -0.017 | -0.124 to 0.090 | 0.055 | 0.757 |
| Intensity | -0.001 | -0.050 to 0.047 | 0.025 | 0.953 |

β – beta coefficient; 95% CI – confidence interval.

**Table S5.** Meta-regression of changes in 24-h mean glucose.

| Covariate | Coefficient  (β) | 95% CI | Standard error | p value |
| --- | --- | --- | --- | --- |
| Intervention duration (weeks) | -0.035 | -0.134 to 0.064 | 0.051 | 0.488 |
| Session duration (min) | -0.013 | -0.040 to 0.067 | 0.028 | 0.624 |
| Work time (s) | -0.002 | -0.006 to 0.003 | 0.002 | 0.457 |
| Rest time (s) | -0.001 | -0.007 to 0.006 | 0.003 | 0.900 |
| Number of bouts | 0.041 | -0.059 to 0.141 | 0.051 | 0.421 |
| Intensity | 0.013 | -0.040 to 0.067 | 0.027 | 0.624 |

β – beta coefficient; 95% CI – confidence interval.

**Table S6.** Meta-regression of changes in fasting glucose.

| Covariate | Coefficient  (β) | 95% CI | Standard error | | p value |  |
| --- | --- | --- | --- | --- | --- | --- |
| Intervention duration (weeks) | 0.064 | -0.236 to 0.365 | 0.153 | 0.675 | | |
| Session duration (min) | -0.001 | -0.073 to 0.070 | 0.036 | 0.969 | | |
| Work time (s) | 0.012 | -0.022 to 0.046 | 0.017 | 0.497 | | |
| Rest time (s) | 0.002 | -0.004 to 0.008 | 0.003 | 0.507 | | |
| Number of bouts | -0.032 | -0.114 to 0.051 | 0.042 | 0.453 | | |
| Intensity | -0.023 | -0.148 to 0.101 | 0.064 | 0.715 | | |

β – beta coefficient; 95% CI – confidence interval


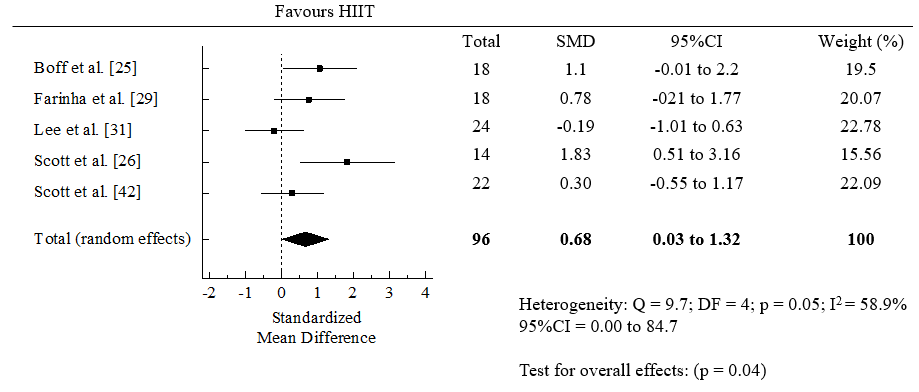


**Figure S1.** Forest plot for change in cardiorespiratory fitness measured by peak oxygen uptake (VO_2peak_, ml/kg/min) before and after (within-group) high-intensity interval training (HIIT).

CI: confidence intervals; DF: degree of freedom; SMD: standardized mean difference.


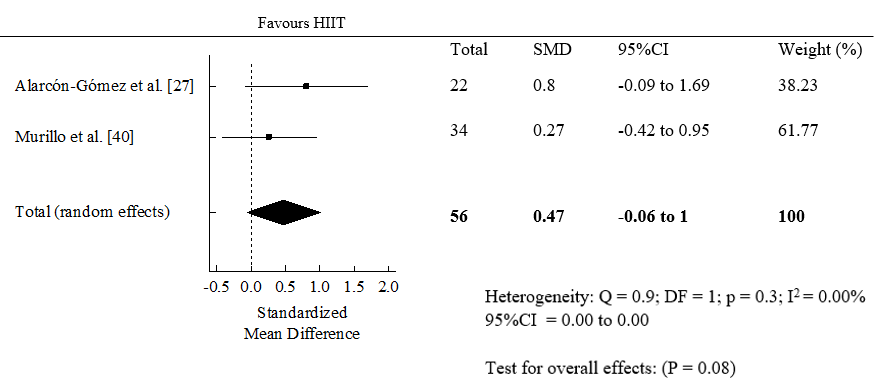


**Figure S2.** Forest plot for change in cardiorespiratory fitness measured by maximum oxygen uptake (VO_2max_, ml/kg/min) before and after (within-group) high-intensity interval training (HIIT).

CI: confidence intervals; DF: degree of freedom; SMD: standardized mean difference.


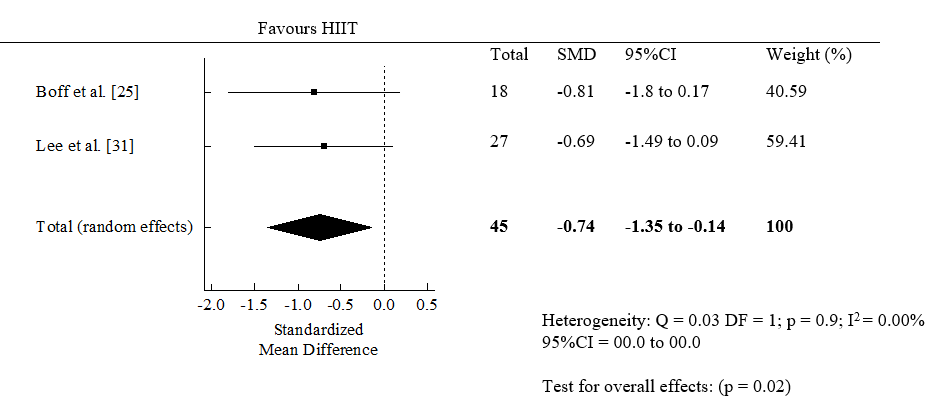


**Figure S3.** Forest plot for change in glycated hemoglobin (HbA1C) between high-intensity interval training (HIIT) and control groups.

CI: confidence intervals; DF: degree of freedom; SMD: standardized mean difference.


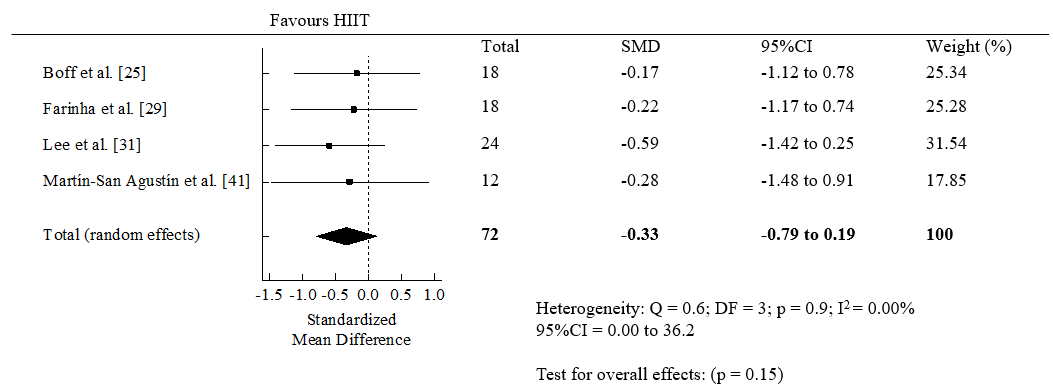


**Figure S4.** Forest plot of within-group effect (> 8 weeks of intervention) of high-intensity interval training (HIIT) on glycated hemoglobin (HbA1C).

CI: confidence intervals; DF: degree of freedom; SMD: standardized mean difference.


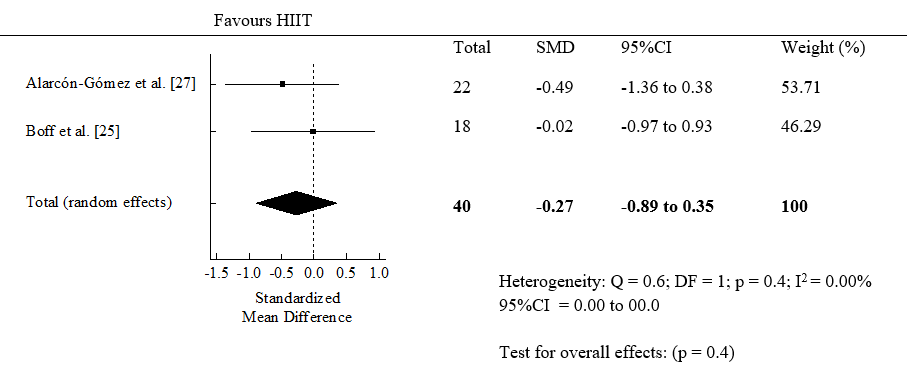


**Figure S5.** Forest plot for change in fasting glucose between high-intensity interval training (HIIT) and control groups.

CI: confidence intervals; DF: degree of freedom; SMD: standardized mean difference.
